# Use of dexmedetomidine repeated subcutaneous administration for balanced anaesthesia in horses

**DOI:** 10.1186/s12917-022-03350-0

**Published:** 2022-07-11

**Authors:** Vanessa Rabbogliatti, Martina Amari, Federica Alessandra Brioschi, Federica Di Cesare, Davide Danilo Zani, Donatella De Zani, Mauro Di Giancamillo, Petra Cagnardi, Giuliano Ravasio

**Affiliations:** Department of Veterinary Medicine and Animal Sciences, Università Degli Studi Di Milano, Milan, Italy

**Keywords:** Balanced anaesthesia, Constant rate infusion, Dexmedetomidine, General anaesthesia, Horse, Recovery, Subcutaneous

## Abstract

**Background:**

A balanced anaesthetic protocol is a common concept in modern veterinary anaesthesia and aims to maintain good intraoperative cardiopulmonary function. In horses, alpha-2-agonists produce sedation and analgesia and have been shown to reduce inhalational anaesthetic requirements when administered intravenously. Furthermore, these drugs can improve recovery quality. Preliminary investigations of subcutaneous dexmedetomidine administration in humans demonstrated a reduced haemodynamic impact if compared with the intravenous route suggesting that dexmedetomidine is adequately absorbed with both administration routes. The aim of the study was to compare two different dexmedetomidine (DEX) administration routes: intravenous constant rate infusion (CRI) versus repeated subcutaneous (SC) injections on cardiopulmonary function and recovery in anaesthetized horses.

**Results:**

No significant differences between groups in heart rate and systolic arterial pressure were detected. A significantly higher mean and diastolic arterial pressure were detected in the SC group at T25 (*p* = 0.04; *p* = 0.02), T75 (*p* = 0.02; *p* = 0.009), and T85 (*p* = 0.001; *p* = 0.005). In SC group there was a significantly lower dobutamine infusion rate (*p* = 0.03) and a significantly higher urinary output (*p* = 0.02). Moreover, recovery quality was higher (*p* = 0.01).

**Conclusions:**

Cardiopulmonary effects in both groups were comparable and within clinical ranges with less dobutamine requirement in the subcutaneous group. Recovery was of better quality with fewer attempts in horses receiving subcutaneous dexmedetomidine. The present study suggests that intravenous constant rate infusion and subcutaneous repeated administration of dexmedetomidine at indicated dosage can be useful in balanced anaesthesia without any systemic or local adverse effects; moreover, in healthy horses undergoing general anaesthesia, repeated subcutaneous dexmedetomidine administration may be a suitable alternative if constant rate infusion is not feasible.

## Background

General anaesthesia in equine patients carries a higher risk of mortality if compared with humans and small animals [1]. The incidence of peri-anaesthetic death may be partly related to the dose dependent cardiovascular depression induced by inhalation anaesthetics [2]. A balanced anaesthetic protocol with the use of inhalation anaesthetics in combination with short-acting anaesthetic adjuvants is a common concept in modern veterinary anaesthesia [3, 4] and aims to maintain good intraoperative cardiopulmonary function [5]. In horses, alpha-2-agonists produce sedation and analgesia and have been shown to reduce inhalational anaesthetic requirements when administered intravenously as a bolus [6] or as a constant rate infusion (CRI) [7, 8]. Furthermore, these drugs can improve recovery quality [4, 5, 9]. Common side effects of alpha-2-agonists include a dose related bradycardia, arrhythmias, a decrease in cardiac output and an increase in vascular resistance [10, 11].

Dexmedetomidine (DEX), the dextro-rotary and active enantiomer of the racemic mixture medetomidine, is the most potent and selective alpha-2-agonist [12]. Alternative administration routes for DEX have been investigated in human [13, 14] and veterinary patients [15]. Due to its beneficial pharmacological profile, DEX has been suggested to be an ideal agent during equine anaesthesia [16, 17]. The use of a DEX CRI at a rate of 1 and 1.75 μg kg hour^−1^ in isoflurane anaesthetized horses under clinical circumstances produced limited cardiopulmonary side effects, significantly improving recovery qualities compared to isoflurane alone [16].

Preliminary investigations of subcutaneous (SC) DEX administration in humans demonstrated a reduced hemodynamic impact if compared with the intravenous (IV) route [13] suggesting that DEX is adequately absorbed with both administration routes [13, 14].

The main purpose of the present study was to compare the clinical effects of two different DEX administration routes in horses undergoing general anaesthesia: SC, never described in equine veterinary medicine, and CRI. Both routes were expected to be similar in terms of cardiopulmonary effects and recovery duration and quality.

## Results

Thirty horses of different breeds were included in the study: 15 horses (two stallions; seven geldings and six mares) received DEX CRI treatment; 15 horses (one stallion; four geldings and 10 mares) received DEX SC administration.

Age (for group CRI and SC respectively, 9.9 ± 3.0 and 9.7 ± 3.5 years), weight (523 ± 65 and 503 ± 66 kg), type of recumbency and total duration of anaesthesia, defined as time from induction to mechanical ventilation interruption (132 ± 19 and 137 ± 23 min) did not differ significantly between groups. In CRI group 10 horses and in SC group eight horses did not fulfil the pre-anaesthetic sedation criteria according to the adapted Taylor’s et al. (2014) [18] scale and required one supplemental IV dose of 2 μg kg^−1^ of detomidine.

Dexmedetomidine CRI duration was 106 ± 22 min while number of SC administrations were 3 in six horses and 2 in nine horses; time from the last subcutaneous administration and end of anaesthesia was 28 ± 16 min. Heart rate and ABPs were compared for the first 95 min following T0.

**Fig. 1:**
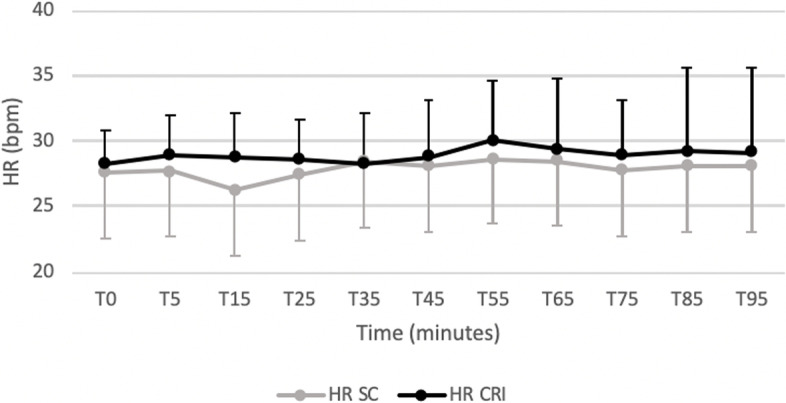
Mean ± standard deviation heart rates (HR) in horses during isoflurane general anaesthesia with dexmedetomidine administered as a constant rate infusion (group CRI) or by repeated subcutaneous administration (group SC)

**Fig. 2:**
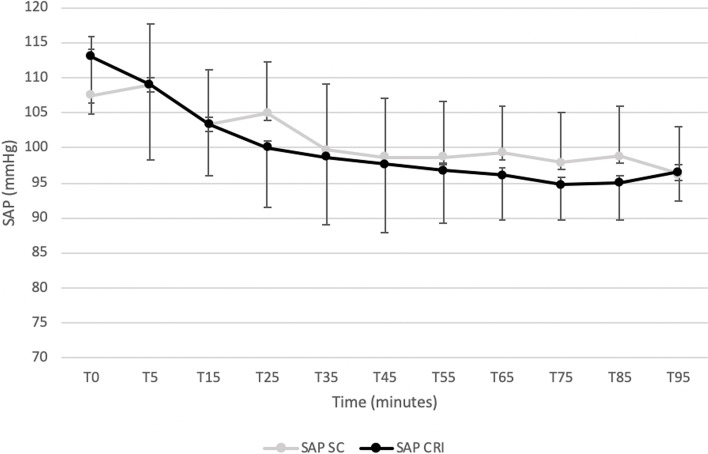
Mean ± standard deviation systolic arterial blood pressures (SAP) in horses during isoflurane general anaesthesia with dexmedetomidine administered as a constant rate infusion (group CRI) or by repeated subcutaneous administration (group SC)

**Fig. 3:**
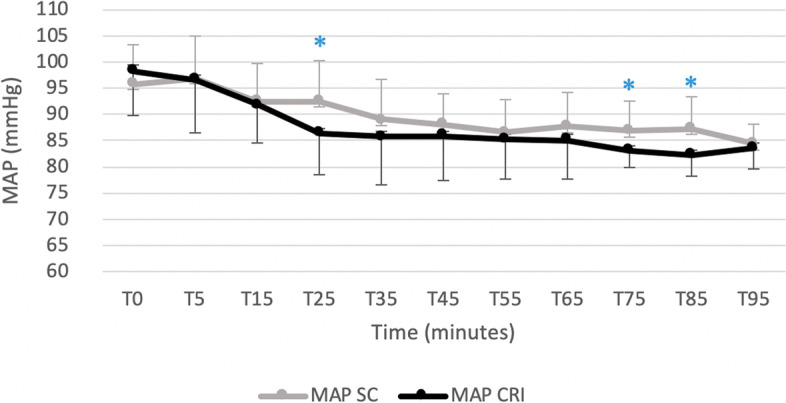
Mean ± standard deviation mean arterial blood pressures (MAP) in horses during isoflurane general anaesthesia with dexmedetomidine administered as a constant rate infusion (group CRI) or by repeated subcutaneous administration (group SC). Significant time points between groups (*p* < 0.05) are indicated with an asterisk (*)

**Fig. 4:**
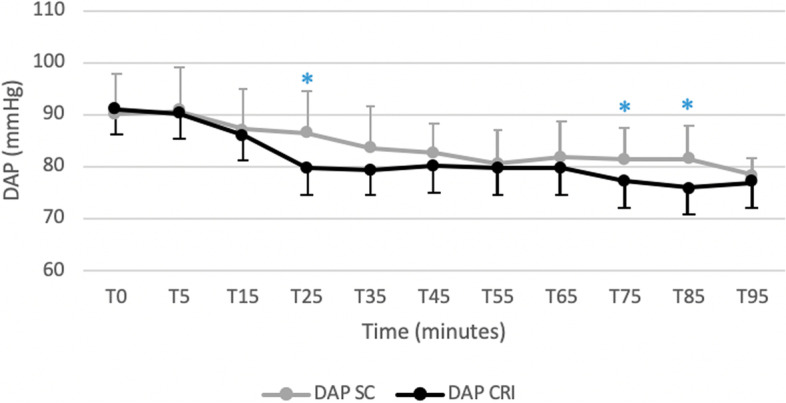
Mean ± standard deviation diastolic arterial blood pressures (DAP) in horses during isoflurane general anaesthesia with dexmedetomidine administered as a constant rate infusion (group CRI) or by repeated subcutaneous administration (group SC). Significant time points between groups (*p* < 0.05) are indicated with an asterisk (*)

Figures 1 and 2 displays cardiopulmonary parameters emphasizing no significative difference between groups in HR and in SAP at all observational time points. Within the SC group a significantly lower HR at T15 compared to all observational time point was recorded. A significantly higher MAP (Fig. 3) and DAP (Fig. 4) were detected in the SC group compared to the CRI group at T25 (for MAP and DAP respectively, *p* = 0.04; *p* = 0.02), T75 (*p* = 0.02; *p* = 0.009) and T85 (*p* = 0.001; *p* = 0.005). A group-independent non-significant decrease over time in SAP, MAP and DAP was noted; moreover, the ABPs significantly decreased in group SC up to T25 while in group CRI up to T15 (Figs. 2, 3 and 4). Differences among arterial blood-gas values are displayed in Table 1.

**Table 1:** Arterial blood-gas values

Parameter	Group	Time points			
		**T0**	**T10**	**T50**	**T90**
**PaCO**_**2**_** mmHg**	**CRI**	40.98 ± 6.82 ^**a**^	38.80 ± 7.34 ^**a**^	38.39 ± 7.29	41.20 ± 7.82
	**SC**	37.39 ± 3.83	37.37 ± 5.44	37.82 ± 3.50	36.61 ± 4.22
**PaO**_**2**_** mmHg**	**CRI**	222.53 ± 26.30^**a−e**^	257.58 ± 31.74 ^**a,e**^	282.94 ± 17.33 ^**b**^	320.37 ± 38.52^a,**c−e**^
	**SC**	229.71 ± 17.93^**b−e**^	265.33 ± 28.43 ^**d,e**^	335.61 ± 53.89 ^**b,d**^	345.79 ± 31.80^**c−e**^
**PaO**_**2**_**/FIO**_**2**_** mmHg**	**CRI**	370.65 ± 26.37^**a−e**^	429.55 ± 51.31^**a,e**^	470.97 ± 28.20^**b**^	533.12 ± 63.51^**c−e**^
	**SC**	391.42 ± 41.69 ^**a−e**^	441.90 ± 46.17 ^**a,e**^	558.39 ± 85.12^**b**^	575.44 ± 52.58^**c−e**^
**pH**	**CRI**	7.42 ± 0.33 ^**a−c**^	7.44 ± 0.06 ^**a,d**^	7.46 ± 0.05 ^**b,d**^	7.44 ± 0.06 ^**c**^
	**SC**	7.45 ± 0.03 ^**a**^	7.47 ± 0.03 ^**a**^	7.47 ± 0.04	7.47 ± 0.03
**Lactate mmol/L**	**CRI**	1.10 ± 0.43	1.06 ± 0.28	1.26 ± 0.47	1.23 ± 0.52
	**SC**	1.42 ± 1.33	1.86 ± 1.94	1.16 ± 0.42 ^#^	1.44 ± 1.03
**Hb g/dL**	**CRI**	11.21 ± 1.55 ^**b,c**^	10.79 ± 1.64 ^**e**^	10.05 ± 1.03 ^**b**^	9.72 ± 1.12 ^**c,e**^
	**SC**	10.56 ± 1.14 ^**b**^	10.59 ± 1.30	11.14 ± 1.03b	10.70 ± 1.16
**Hct %**	**CRI**	33.80 ± 4.57	32.20 ± 4.83	30.55 ± 2.25	29.22 ± 3.53
	**SC**	31.60 ± 3.31	31.80 ± 3.97	33.50 ± 3.18	32.11 ± 3.41
**BE mmol/L**	**CRI**	2.21 ± 2.32	1.97 ± 2.03 ^**e**^	2.68 ± 1.54	3.51 ± 1.98^**e**^
	**SC**	1.09 ± 4.53 ^**#**^	-0.78 ± 3.13 ^**#**^	-0.19 ± 2.85 ^**#**^	1.33 ± 2.49 ^**#**^
**Na**^**+**^	**CRI**	134. 46 ± 1.74 ^**a−c**^	135.05 ± 1.47 ^**a**^	135.53 ± 2.17 ^**b,f**^	136.41 ± 3.16 ^**c,f**^
	**SC**	134.46 ± 2.02 ^**b,c**^	134.80 ± 2.27 ^**d,e**^	135.97 ± 2.00 ^**b,d,f**^	136.60 ± 1.89 ^**c,e,f**^
**K**^**+**^	**CRI**	3.60 ± 0.23	3.46 ± 0.28	3.51 ± 0.27	3.62 ± 0.61
	**SC**	3.75 ± 0.26 *	3.71 ± 0.35	3.60 ± 0.26	3.70 ± 0.27
**Ca**^**2+**^	**CRI**	1.43 ± 0.04 ^**b,c**^	1.40 ± 0.06	1.39 ± 0.05 ^**b**^	1.37 ± 0.07 ^**c**^
	**SC**	1.45 ± 0.08	1.43 ± 0.05 ^**e**^	1.42 ± 0.05^**f**^	1.40 ± 0.05 ^**e,f**^
**Cl**^**−**^	**CRI**	102.51 ± 1.76	103.14 ± 2.38	103.05 ± 2.05	102.47 ± 3.45
	**SC**	105.51 ± 2.84	105.16 ± 2.74	104.66 ± 1.86	104.11 ± 1.52
**Glu mg/dL**	**CRI**	125.89 ± 20.87 ^**b,c**^	129.67 ± 29.24 ^**d,e**^	183.00 ± 41.36^**b,d,f**^	221.50 ± 46.67 ^**c,e,f**^
	**SC**	133.00 ± 26.36^**a−c**^	145.86 ± 23.82^**a,d,e**^	199.56 ± 39.52 ^**b,d**^	226.43 ± 39.63 ^**c,e**^
**Creatinine** **mg/dL**	**CRI**	1.35 ± 0.40	1.36 ± 0.44	1.34 ± 0.44	1.40 ± 0.48
	**SC**	1.23 ± 0.59	1.25 ± 0.19	1.28 ± 0.19	1.17 ± 0.21
**Urea** **mg/dL**	**CRI**	27.61 ± 3.93	27.26 ± 4.44	27.16 ± 3.53	27.20 ± 3.72
	**SC**	21.22 ± 11.60	25.40 ± 5.83	23.30 ± 8.17	27.87 ± 3.80

Mean ± standard deviation of arterial blood-gas values, arterial oxygen and carbon dioxide partial pressures (PaCO_2_, PaO_2_), ratio of arterial partial pressure of oxygen to fraction of inspired oxygen (pO_2_/FIO_2_), arterial pH, arterial lactate, glucose and haemoglobin concentrations, hematocrit, arterial base excess, electrolytes, urea and creatinine in horses during isoflurane general anaesthesia with dexmedetomidine administered as a constant rate infusion (group CRI) or by repeated subcutaneous administration (group SC). Significant differences between groups (*p* < 0.05) at the same time point: # significantly lower in SC; * significantly higher in SC group. Significant differences (*p* < 0.05) within group over time: ^a^T0 Vs T10; ^b^T0 Vs T50; ^c^T0 Vs T90; ^d^T10 Vs T50; ^e^T10 Vs T90; ^f^T50 Vs T90

Preliminary results regarding DEX serum quantification showed a range of maximum concentration of 0.50 – 2.27 ng ml^−1^ in SC group and of 0.39 – 1.18 ng ml^−1^ in CRI group.

Six out of 15 horses in CRI group required one rescue ketamine bolus during general anaesthesia and two out of 15 horses in SC group required one rescue ketamine bolus. No horses in both groups required thiopental administration. There was a significantly lower dobutamine infusion rate in the SC group (CRI: 0.8 ± 0.4 μg kg minute^−1^ vs SC: 0.6 ± 0.2 μg kg minute^−1^; (*p* = 0.03). Urine output was significantly higher in SC group (CRI: 6.7 ± 2.5 ml kg hour^−1^ vs SC 8.8 ± 2.8 ml kg hour^−1^; *p* = 0.02). No change in rate or type of fluid administration was required.

Recovery times did not differ significantly between groups; also number of attempts to sternal recumbency did not significantly differ among groups (Table 2); the number of attempts to stand was significantly higher in CRI group (*p* = 0.03). Based on the simple descriptive scale [19] recovery score quality was higher in SC group (*p* = 0.01) (Table 2).

**Table 2:** Recovery timing and score

	**Group CRI**	**Group SC**	
Time To Spontaneous Ventilation (Min)	5.7 ± 3.6	4.4 ± 1.9	*p* = 0.09
Time To Extubation (Min)	10.6 ± 4.3	9.4 ± 2.9	*p* = 0.17
Time Between Spontaneous Ventilation And Extubation (Min)	4.9 ± 2.1	5.1 ± 1.9	*p* = 0.43
Time To Sternal Recumbency (Min)	40.2 ± 13.6	47.0 ± 9.8	*p* = 0.09
Attempts To Sternal Recumbency (N°)	1.8 ± 0.8	1.4 ± 0.6	*p* = 0.34
Time To Standing (Min)	51.7 ± 14.4	57.5 ± 10.3	*p* = 0.12
Attempts To Standing (N°)	1.8 ± 0.8	1.4 ± 0.6	*p* = 0.03
Recovery Score (Young & Taylor)	4.2 ± 1.0	4.7 ± 0.5	*p* = 0.01

Mean ± standard deviation of times in minutes (min) to spontaneous ventilation, to extubation, between spontaneous ventilation and extubation and time to sternal recumbency and standing. Number (n°) of attempts to sternal recumbency and standing. Final recovery score evaluated with a simple descriptive scale. A score of 5 represented a recovery with no ataxia, no struggling, standing up at first attempt as fully conscious; while, a score of 0 was used for a very violent (wall of death), self-inflicted injury, prolonged struggling or unable to stand 2 h after the end of anaesthesia [19]. Parameters were recorded during the recovery phase in horses undergoing isoflurane general anaesthesia with dexmedetomidine administered as a constant rate infusion (group CRI) or by repeated subcutaneous administration (group SC). (*p* < 0.05)

No horses required additional rescue bolus of DEX during recovery. During the 10 days of follow-up period no systemic effects or local signs of irritation, inflammation or swelling at the injection site were detected.

## Discussion

Among the different drug administration routes, subcutaneous injection is performed within the fatty layer of the subcutaneous tissue just beneath the skin. As subcutaneous tissue has few blood vessels, the injected drug is diffused very slowly at a sustained rate of absorption [20].

Due to lack of studies on SC DEX administration in companion animals, the authors previously decided to evaluate a dose of 1 μg kg^−1^ based on human literature [13, 14]. However, this dose was not sufficient to produce a stable anaesthetic plane similar to CRI at 1 μg kg hour^−1^ (authors unpublished data), therefore DEX dose was increased at 2 μg kg^−1^ maintaining the 60-min interval. Indeed, a drawback of subcutaneous drugs administration is the incomplete bioavailability of the injected molecule, which can range widely from 50 to 80% [21, 22]. The incomplete bioavailability typically results in the need for a higher dose for subcutaneous injection than for intravenous infusions [23]. Moreover, as reported also for other alternative routes of administrations, many factors may contribute to affect drug bioavailability, such as the patient’s positioning, other drugs’ co-administration, and the variations in physiological parameters (e.g. HR, ABP, and body temperature) due to general anaesthesia [24]. In this study, the patient’s positioning and thermal dispersion were controlled, but being the pharmacokinetic properties of DEX after subcutaneous administration unpredictable, the authors' decided to double the dosage compared to CRI.

A loading dose of DEX by IV route could also be included within the protocol to achieve a faster steady-state condition. The authors’ decision to not administer an initial DEX bolus was based on the possible major cardiovascular effects that a loading dose by IV route could have caused [17], overshadowing the cardiovascular effects caused by SC and CRI DEX and the potential differences between the two administration routes. In clinical settings, the alpha-2-agonist given for premedication is usually considered as the loading dose [17], even if differs from the alpha-2-agonist given by CRI [25].

Both administration routes induced similar cardiopulmonary effects. Although statistical differences were detected at different time points for HR, ABPs and in some blood gas parameters, these differences were minimal and of limited importance in healthy horses. Heart rate was not significantly different between groups and neither within groups in time except for a significantly lower HR within the SC group at T15 (HR 26 ± 5 bpm) compared to all observational time points. This result could be attributable to a peak concentrations of DEX at 15 min that has been reported by Uusalo and colleagues (2018) after SC administration in human medicine [13]. No periods of severe bradycardia (HR < 20 bpm) [26] were observed.

Within both groups, a decrease over time of ABPs was found. This reduction was significant in group CRI up to T15 and in group SC up to T25. This finding could be correlated to the gradually waning effect of the detomidine, that has been shown to be short-lasting after IV administration, with an elimination half-life of 30 min [27] in combination with a hypotensive effect caused by inhaled anaesthetics [28] and the lack of a fluid supplementation based on the actual urinary loss. However, these values were always above to those considered acceptable during horse anaesthesia and in part this may be explained by the use of a dobutamine CRI to maintain MAP above 70 mmHg. Interestingly, a significantly higher MAP and DAP were detected in the SC group compared to the CRI group at T25, T75 and T85. These time points could represent peaks of absorption of DEX administered by SC route. Moreover, the ABPs values tended in general to be higher in the SC group probably due to a higher DEX dose administered in comparison with the CRI group. The vasoconstriction induced by the administration of DEX [29] could have contributed to maintain higher ABPs values in the SC group despite a significantly lower dose of dobutamine administered (CRI: 0.8 ± 0.4 μg kg minute^−1^; SC: 0.6 ± 0.2 μg kg minute^−1^).

Due to the lack of information in horses on the pharmacokinetic profile of DEX after SC administration, it is not possible to state whether the absorption peak in this species is the same as in humans [13]; nevertheless, it can be assumed from the results of this study that the pharmacodynamics effects are clinically evident in horses within 25 min after the first SC administration. A future study on the pharmacokinetic of DEX administered by SC route is ongoing. Preliminary results demonstrated that the serum maximum concentration reached in both groups are similar or higher to those reported by Bettembourg and colleagues (2019) that also observed the clinical effects of DEX in horses under general anaesthesia [30].

In this study, urinary output was significantly higher in group SC compared to CRI group (CRI: 6.7 ± 2.5 ml kg hour^−1^; SC 8.8 ± 2.8 ml kg hour^−1^). Alpha-2- agonists are known to increase urine production in awake [31] and anaesthetized healthy equids [6, 32], mainly due to a reduced secretion rate of arginine vasopressin and due to hyperglycemia caused by hypoinsulinemia [31, 32]. The difference among groups in urine output is probably related to the higher ABPs and glycaemia in the SC group.

Blood glucose significantly increased in both groups. This result is in according to what obtained by Ringer and colleagues (2013), in which plasma glucose concentration continued to increase during CRIs of xylazine and romifidine and tended to decrease once apha-2- agonists infusions were stopped [33]. Through the binding in the pancreatic cells, apha-2- agonists decrease insulin secretion that causes hyperglycaemia in adult horses [31]. Interestingly, even if DEX SC administration was repeated, no glycaemic peaks have developed as it happens following an IV bolus of dexmedetomidine [34], showing that repeated SC administration can produced clinical effects comparable with IV CRI.

To reduce the potential risk of post-anaesthetic myopathy, the maintenance of both adequate cardiac performance and oxygen delivery is imperative [35]. In this study, patients were all positioned in left lateral recumbency and mechanical ventilation was used. Results from arterial blood gas analysis showed no significative differences between groups in PaO_2_, PaCO_2_ and PaO_2_/FIO_2_ at all time points. Besides, these values remained always within normal ranges in both groups during the entire anaesthesia period. General anaesthesia was well maintained in both groups, a greater stability of the anaesthetic depth was found in the SC group demonstrated by the need of ketamine rescue in only two horses compared to the six horses in the CRI group.

Recovery from general anaesthesia in the horse is a difficult period to manage [1, 19]. To ensure greater safety during this critical phase, alpha-2-agonists have been used to improve recovery quality in horses after inhalant anaesthesia [36]. Since during standing most traumatic injuries can occur, it is important to reduce the number of attempts to stand. In our study recovery was significantly more successful with fewer attempts to stand in horses administered with SC DEX compared to CRI.

Santos and colleagues (2003) compared recovery times in isoflurane anesthetized horses after administration of xylazine, romifidine and detomidine during recovery [36]. Interestingly, although no rescue DEX was administered during recovery in our study, recovery times were longer when compared with all groups in Santos’s study, but similar to those receiving DEX or medetomidine [9]. Recovery time of our treatments were prolonged (CRI: 51.7 ± 14.4 min; SC: 57,5 ± 10.3 min) but within times suggested in literature [37]. No significant difference in time to standing was found between groups, despite the average time from the last SC administration and the end of anaesthesia was 28 ± 16 min. Probably also this finding reflects a slow absorption and a prolonged steady state plasma concentration of DEX given by SC injection. Both groups show a good recovery quality although there was a significantly higher recovery score in the SC group (CRI: 4.2 ± 1.0; SC: 4.7 ± 0.5; *p* = 0.01).

The prolonged recovery times together with the good recovery quality suggest that both DEX administration routes provide good sedation that extends during the recovery phase permitting the elimination of inhaled anaesthetic and producing a slow and quite recovery from general anaesthesia in these patients.

The design of the present clinical study imposed several limitations. The results obtained may have been influenced by the simultaneous administration of other anaesthetic drugs used for premedication and induction. The authors decided to maintain a standard isoflurane end-tidal and fluid therapy to actually assess the possible influence of the DEX different routes of administration on cardiopulmonary parameters, dobutamine infusion rate and urinary output. Another limit of the study is the utilization of detomidine for patient premedication before induction; this could be a variable to considerer when evaluating the first anaesthesia period. Another limitation may be that the DEX serum concentrations reached were not fully equipotent for all the clinical variables evaluated, therefore some findings may have been influenced by the different DEX dose administered SC and through CRI. Further studies are essential to evaluate SC administration in patients undergoing surgery; however, the inclusion of horses undergoing magnetic resonance examination has allowed to partially standardize the procedure permitting a better comparison between the two administration routes.

## Conclusions

The intravenous CRI and subcutaneous administration of dexmedetomidine at indicated dosage can be useful in balanced anaesthesia without any systemic or local adverse effects. Cardiopulmonary effects in both groups were comparable and within clinical ranges with less dobutamine requirement in the SC group. Recovery was of better quality with less attempts in horses receiving SC DEX compared to those treated with CRI. In conclusion, in healthy horses undergoing general anaesthesia, the SC DEX may be a suitable alternative if CRI is not feasible.

## Methods

### Animals and Instrumentation

The study was approved by the Institutional Ethical Committee for Animal Care of the University of Milan (OPBA 17_2020). All procedures were carried out in accordance with the relevant guidelines and regulations and the study was carried out in compliance with the ARRIVE guidelines. Owner informed written consent was obtained. Thirty client-owned, non-food-producing horses of various breeds presented for magnetic resonance examination under general anaesthesia were included in this randomized controlled trial study. Inclusion criteria were body weight (> 200 kg), age (2—20 years), physical status American Society of Anesthesiologists (ASA) I or II based on physical examination and on blood work.

In case of intra-anaesthetic complications such as severe hypotension or bradycardia, not sufficient depth of anaesthesia with horses requiring more than 2 ketamine / thiopental boluses, need to modify rate and/or fluid administration, the DEX administration was interrupted and patients excluded from the study. Moreover, in case of a single SC DEX administration horses were excluded.

Food but not water was withheld for 12 h before general anaesthesia. Horses were randomly assigned to receive DEX either by IV CRI (CRI group) or by repeated SC administration (SC group) (www.randomizer.org). All anaesthetic procedures were performed by the same experienced anaesthetist while the recovery phase was evaluated and scored by a second experienced anaesthetist blind to the treatment. After clipping and skin disinfection, a 14-gauge × 64 mm (Surflo®, Terumo Europe N.V., Belgium) jugular catheter was placed following desensitization of the insertion site with 1 mL of Lidocaine 2% (Lidocaina 2%, ECUPHAR ITALIA S.r.l, Italy). Each horse received IV acepromazine (Prequillan 1%, FATRO S.p.A., Italy) 0.03 mg kg^−1^ then, the mouth was rinsed, and horseshoes removed. After 15 min from acepromazine administration horses were sedated with detomidine (Domosedan, Orion Pharma S.r.l., Italy) at 10 μg kg^−1^ IV and sedation score was assessed. Sedation was considered adequate when criteria of the scale used by Sacks and colleagues (2017) adapted from Taylor et al. (2014) were fulfilled [18]. These criteria included head height lower than withers, lower lip atonic and no reaction to stimulation with a pen, when touching the inside of the ears. If one of the conditions were not present five minutes from the detomidine administration, a supplemental dose of detomidine 2 μg kg^−1^ was administered IV. Sedation was then re-evaluated after five minutes and, if necessary, this procedure was repeated. Induction of anesthesia was achieved 10 min after the last detomidine administration with IV diazepam (Ziapam, Laboratoire TVM, France) 0.08 mg kg^−1^ followed by IV ketamine (Ketavet 100, MSD Animal Health S.r.l., Italy) 2.5 mg kg^−1^.

Once the horses were recumbent and orotracheal intubation was obtained (silicon tubes, internal diameter 24–28 mm), they were hoisted onto a padded (20 cm) MRI-dedicated table and moved into the faraday cage. All patients were in left lateral recumbency. Mechanical ventilation (Mallard 2800C-P MRI compatible, AB Medical Technologies Inc., USA) was immediately initiated with a tidal volume of 15 ml kg^−1^, peak inspiratory pressure close to 25 cmH_2_O, positive end-expiratory pressure of 5 cmH_2_O; inspiratory time was set at 2.0 s and expiratory time was modified in order to change the respiratory rate and maintain normocapnia (35–45 mm Hg). Anaesthesia was maintained with isoflurane (Isoflo, Zoetis Italia S.r.l., Italy) delivered in a mixture of oxygen (O_2_) and air, so as to maintain the inspired O_2_ fraction (FiO_2_) between 60—65%; isoflurane vaporized settings were adjusted to maintain an end-tidal of 1.3% (Datex Ohmeda S5, GE Healthcare, Italy).

If nystagmus or fighting against the ventilator occurred, IV ketamine at 0.1 mg kg^−1^ was delivered; in case of sudden movements, IV thiopental (Pentothal sodium, MSD Animal Health, Italy) 0.5 mg kg^−1^ was administered.

Invasive arterial blood pressures (ABPs) were monitored via a 20-gauge × 51 mm catheter (Surflo®, Terumo Europe N.V., Belgium) inserted into the transverse facial artery. The catheter was connected to a pressure transducer (Datex Ohmeda S5, GE Healthcare, Italy), placed at the level of the heart with the scapulohumeral joint as a reference point and zeroed to ambient pressure. Heart rate (HR) was continuously monitored through pulse palpation. An urinary catheter was placed to empty the bladder and it was then left in position to evaluate urinary output during dexmedetomidine administration.

At 15 min from induction, once patient preparation and instrumentation was achieved, DEX administration was initiated (T0).

### Experimental design

At T0 horses in CRI group received DEX 1 μg kg hour^−1^ IV constant rate infusion, diluted at 20 μg ml^−1^ in saline solution (NaCl 0.9%, B Braun, Germany) whereas horses in SC group received DEX 2 μg kg^−1^ by subcutaneous injection repeated every hour. Constant rate infusion was delivered by a syringe pump (Mindray SK-500II, Mindray Medical Italy S.r.l., Italy) while SC administration was performed at the level of the neck using a 2.5 mL syringe with a 22-gauge × 32 mm needle (RAYS, SPA, Italy).

Ringer's lactated solution (Ringer Lattato, S.A.L.F., Italy) at 2 ml kg hour^−1^ was administered IV throughout intra-anesthetic period. In order to maintain MAP ≥ 70 mmHg, dobutamine (Miozac, Fisiopharma S.r.l., Italy) was administered IV by an infusion pump (Mindray SK-500II, Mindray Medical Italy S.r.l., Italy) with a starting dose of 1.25 μg kg minute^−1^ and the rate was increased in 0.1 μg kg minute^−1^ every five minutes if needed. Dobutamine rate reduction / interruption was also based on IBP. In case of MAP between 75 and 90 mmHg the rate was reduced of 0.1 μg kg minute^−1^ for five minutes and evaluated again. In case of MAP > 90 mmHg the infusion was stopped for five minute and then assessed. Heart rate, systolic arterial pressure (SAP), mean arterial pressure (MAP) and diastolic arterial pressure (DAP) were recorded from T0, at five minutes (T5) and thereafter at ten-minutes intervals. Collection of arterial blood samples was achieved by a 20-gauge × 51 mm catheter (Surflo®, Terumo Europe N.V., Belgium) placed in the metatarsal artery; samples (approx. 2.5 mL each) were anaerobically withdrawn using pre-heparinized syringes and immediately analyzed (Stat Profile® pHOx® Ultra, Nova Biomedical Italia S.r.l., Italy) at T0 and then at ten (T10), fifty (T50) and ninety (T90) minutes. Analysis included pH, arterial oxygen and carbon dioxide partial pressures (PaO_2_, PaCO_2_), hemoglobin and hematocrit, base excess (BE), electrolytes (Na^+^, K^+^, Ca^2+^, Cl^−^), arterial lactate, glucose, creatinine and urea. In addition, serial blood samples were collected for DEX serum quantification according to a validated method described by Cagnardi et al. (2017) [38] with some modifications. The comparison between CRI and SC pharmacokinetic profiles will not be illustrated in this study as they will be described in a separate publication.

The number of rescues of ketamine and thiopental together with the mean cumulative dose of dobutamine administered and urinary output was finally calculated for each horse according to the individual body weight and the duration of anaesthesia.

At the end of the magnetic resonance examination DEX CRI was interrupted, and in both groups mechanical ventilation was stopped, and the recovery timing started. Horses were moved to a padded recovery stall, in a quiet and soft lights environment.

Adequate ventilation was guaranteed using a demand valve (3 breaths per minute in 100% O_2_) until the horses started to breathe spontaneously then, O_2_ flow-by (15 L minute^−1^) was administered initially through the endotracheal tube and nasally after extubation. If horses either moved or showed excessive nystagmus and were in danger of an untimely recovery, DEX at 1 μg kg^−1^ was administered IV and the patients were excluded from the study. The trachea was extubated once the horses regained their swallow reflex. Anesthesiologist evaluated the degree of respiratory effort and, if considered excessive, a nasal tube was passed. Horses were allowed to recover without assistance. Times to extubation, to sternal recumbency, and time to standing were recorded; also, number of attempts to achieve sternal recumbency and standing position were noted.

An experienced blind anaesthetist assessed the recovery quality. It was graded on a standard scoring 5-point simple descriptive scale [19] with a score of 5 representing a recovery with no ataxia, no struggling, standing up at first attempt as fully conscious; while, a score of 0 was used for a very violent (wall of death), self-inflicted injury, prolonged struggling or unable to stand two hours after the end of anaesthesia. A follow-up period of 10 days was planned to evaluate any systemic and local side effects.

### Statistical analysis

Sample size calculation indicated that a minimum of 26 patients (*n*° = 13 per group) were sufficient to obtain power values > 90% with an effect size of 0.5 (medium) at an alpha level of 0.05.

The Shapiro–Wilk test was used to verify the normality of distribution of data. Data with normal distribution are presented as mean ± SD and were analyzed by repeated-measures one-way analysis of variance (ANOVA) with post hoc Bonferroni multiple comparisons test to evaluate the effects between all time points within each treatment and using one-way ANOVA with post hoc Tukey–Kramer’s test to identify differences among the treatments at each time point. Data with non-normal distribution are presented as median with range (minimum, maximum) and were analyzed using Friedman tests for within-treatment analyses and Kruskal–Wallis test for between-treatment comparisons, with post hoc Dunn’s multiple comparisons test, respectively. Significance was established at *P* < 0.05. All data were analyzed using commercially available software (PASW 18.0—SPSS Inc, USA).

## Data Availability

The datasets used and/or analyzed during the current study supporting our results are included in the article. Row data are available from the corresponding author upon reasonable request.
